# Transforming health professions’ education through in-country collaboration: examining the consortia among African medical schools catalyzed by the Medical Education Partnership Initiative

**DOI:** 10.1186/1478-4491-13-1

**Published:** 2015-01-14

**Authors:** Zohray M Talib, Elsie Kiguli-Malwadde, Hannah Wohltjen, Miliard Derbew, Yakub Mulla, David Olaleye, Nelson Sewankambo

**Affiliations:** The George Washington University School of Medicine and Health Sciences, Washington, DC USA; African Centre for Global Health and Social Transformation, Kampala, Uganda; Department of Health Policy, the George Washington University, Washington, DC USA; College of Health Sciences, Addis Ababa University, Addis Ababa, Ethiopia; University of Zambia School of Medicine, Lusaka, Zambia; Faculty Basic Medical Science, College of Medicine, University of Ibadan, Ibadan, Nigeria; Makerere University College of Health Sciences, Kampala, Uganda

**Keywords:** Medical education, South-south partnerships, Workforce scale-up

## Abstract

**Background:**

African medical schools have historically turned to northern partners for technical assistance and resources to strengthen their education and research programmes. In 2010, this paradigm shifted when the United States Government brought forward unprecedented resources to support African medical schools. The grant, entitled the Medical Education Partnership Initiative (MEPI) triggered a number of south-south collaborations between medical schools in Africa. This paper examines the goals of these partnerships and their impact on medical education and health workforce planning.

**Methods:**

Semi-structured interviews were conducted with the Principal Investigators of the first four MEPI programmes that formed an in-country consortium. These interviews were recorded, transcribed and coded to identify common themes.

**Results:**

All of the consortia have prioritized efforts to increase the quality of medical education, support new schools in-country and strengthen relations with government. These in-country partnerships have enabled schools to pool and mobilize limited resources creatively and generate locally-relevant curricula based on best-practices. The established schools are helping new schools by training faculty and using grant funds to purchase learning materials for their students. The consortia have strengthened the dialogue between academia and policy-makers enabling evidence-based health workforce planning. All of the partnerships are expected to last well beyond the MEPI grant as a result of local ownership and institutionalization of collaborative activities.

**Conclusions:**

The consortia described in this paper demonstrate a paradigm shift in the relationship between medical schools in four African countries. While schools in Africa have historically worked in silos, competing for limited resources, MEPI funding that was leveraged to form in-country partnerships has created a culture of collaboration, overriding the history of competition. The positive impact on the quality and efficiency of health workforce training suggests that future funding for global health education should prioritize such south-south collaborations.

## Background

In recent years, the demands on African medical schools have intensified [1, 2]. The critical shortage of healthcare workers has driven many African governments to mandate significant increases in medical school enrollment and improvements in the quality of training [3]. Recognizing the gap between the ideal health workforce and the capacity of training institutions, a landmark report called for critical reforms in global health education [4]. One of the proposed institutional reforms is the establishment of ‘networks, alliances and consortia’ as a strategy for schools to collaborate and share resources [4]. Historically, African medical schools worked either alone or with northern partners to produce a capable health workforce in environments with limited resources. Kolars *et al.* highlight the challenges of north-south partnerships, which include the dominance of the northern partners’ agenda, the inadvertent contribution to brain-drain, a low degree of sustainability due to external sources of funding, and a focus on short-term goals rather than long-term capacity-building interventions [5]. Reports of south-south partnerships are limited but include the Collaboration for Health Equity in Education and Research (CHEER) among eight Health Science Faculties in South Africa focusing on community-based education and the Consortium of New Southern African Medical Schools (CONSAMS), which recently formed among five relatively new medical schools [6, 7]. Thus, while medical schools in Africa face similar challenges, there has been relatively little networking and collaboration among schools to date.

In 2010, the United States Government directed unprecedented resources to support African medical schools in meeting their training needs and triggered a number of collaborations, including those between south-south partners in Africa [8, 9]. The Medical Education Partnership Initiative (MEPI) distributed $150 million to 13 medical schools in 12 sub-Saharan African countries. The thematic focus of MEPI is on improving the quality, quantity, and in-country retention of medical graduates along with building research capacity of African medical schools. The initiative emphasizes country-ownership, allowing recipient schools to tailor their grant activities to local needs. While partnerships were encouraged but not required, several schools applied as in-country consortia [10]. Despite the different historical contexts in which these consortia were formed, they all redefine the relationships among in-country medical schools.

For all consortia, the MEPI funding was awarded to one medical school as the primary grantee with partner schools as subcontractors. In Uganda, the MEPI grant was awarded to Makerere University, which partnered with all four schools in the country, including one private medical school. The consortium is now called the Medical Education for Equitable Services to All Ugandans (MESAU) consortium. In Ethiopia, the primary grantee is Addis Ababa University and the consortium includes two new public medical schools and a military school. In Nigeria, the lead school for the MEPI award is the University of Ibadan, which formed the Medical Education Partnership Initiative in Nigeria (MEPIN) consortium with six in-country public schools. The University of Zambia did not initially apply for MEPI funds with in-country medical school partners but soon after the grant was awarded, the countrywide consortium formed with one public and two new private medical schools.

This paper describes these four consortia within the Medical Education Partnership Initiative and examines their common goals, their approach to sharing limited resources, their collaborative efforts to overcome common challenges, and ultimately how they are improving the quality of medical education and health workforce planning. Given the paucity of south-south collaboration among medical schools, this paper also seeks to describe the challenges and key enablers that have allowed these partnerships to succeed.

## Methods

Grounded theory was used as a qualitative framework for this study. This approach was selected because it emphasizes an inductive approach to data collection and analysis and provides an opportunity to discover and compare nuances in individual schools’ experiences with consortia partnerships. The study team used semi-structured interviews to explore these experiences and the role of consortia in medical education scale-up and workforce planning. Key informants for the semi-structured interviews were selected from the first four MEPI programmes to form in-country consortia. Three programmes (Nigeria, Ethiopia and Uganda) formed their consortia at the time of applying for MEPI while Zambia formed their consortia soon after they received the award. In subsequent years, other MEPI programmes formed in-country consortia but the decision was made to only include the first 4 in this study to ensure that the programmes participating had at least 2 years of collaborative activities to reflect on during the interview. The interviewees were the four Principal Investigators (PIs) for the four selected MEPI programmes. The PIs are responsible for overseeing the MEPI-funded activities of their consortia. The team that developed the interview guide and analyzed the data included ZT, HW and EK who are all part of the MEPI Coordinating Centre and not attached to any of the four schools examined in this study. The lead author (ZT) developed a semistructured interview guide with input from (HW and EK). Interviewees were asked to openly reflect on different aspects of the consortia including the benefits, challenges, sustainability, impact on national health workforce goals, and influence of MEPI on their establishment. All four semi-structured interviews were conducted by ZT in English, using Skype and recorded using MP3 Skype Recorder. Consent was obtained by ZT at the start of each interview. The 4 interviews lasted between 60 and 80 minutes. HW transcribed the interviews that were then entered into QSR-NVivo (Burlington, MA, USA) for data analysis. A codebook paralleling the themes of the interview questions was developed, and two members of the research team (ZT and EK) independently coded the transcripts. Additional codes were included in the codebook as more themes emerged from the transcripts. Manuscript preparation was undertaken collaboratively with the PI interviewees who validated the themes that emerged from the coding and verified the accuracy of the data used. The MEPI Coordinating Centre provided data included in Table 1 that was obtained from the results of its 2013 MEPI annual survey and verified by the four MEPI PIs interviewed for the study. Ethical approval for this study was deemed unnecessary by the George Washington University Institutional Review Board (IRB).

**Table 1:** **Consortia and their membership: characteristics of each school**

	School	Location	Year established	Ownership	Number of undergraduate students	Number of postgraduate trainees	Number of faculty
Ethiopia	Addis Ababa University College of Health Sciences	Addis Ababa	1964	Public	1,297	526	342
	Defense University College	Debre Zeit	2007	Public	58	0	36
	Haramaya University School of Medicine	Dire Dawa	2007	Public	902	0	51
	Hawassa University	Hawassa	2003	Public	1,026	71	79
Nigeria	University of Ibadan College of Medicine	Ibadan	1948	Public	1,419	1214	361
	Ahmadu Bello University, Faculty of Medicine	Zaria	1967	Public	551	310	266
	University of Jos, Faculty of Medical Sciences	Jos	1978	Public	2,766	205	149
	College of Medicine University of Lagos, Nigeria	Lagos	1962	Public	1,022	500	441
	University of Maiduguri College of Medical Sciences	Maiduguri	1978	Public	857	85	130
	University of Nigeria College of Medicine	Enugu	1970	Public	1,261	89	228
Uganda	Makerere University College of Health Sciences	Kampala	1924	Public	621	279	327
	Busitema University, Faculty of Health Sciences	Mbale	2013	Public	53	0	22
	Gulu University Faculty of Medicine	Gulu	2004	Public	346	7	27
	Kampala International University W.C. School of Medicine	Bushenyi Ishaka	2004	Private	1,136	0	109
	Mbarara University of Science and Technology, Faculty of Medicine	Mbarara	1989	Public	364	33	89
Zambia	University of Zambia School of Medicine	Lusaka	1966	Public	495	333	86
	Cavendish University School of Medicine	Lusaka	2009	Private	--	--	--
	Copperbelt University School of Medicine	Ndola	2011	Public	225	10	47
	Lusaka Apex Medical University	Lusaka	2010	Private	97	10	40

-- = information not provided on Round 3 survey.

## Results and discussion

### Established schools lead the way in forming consortia

All four consortia are led by well-established public medical schools. Three of the consortia include new schools and two of the consortia include a private medical school. In Uganda, the partnership was initiated by the oldest medical school in the country, Makerere University, which reached out to all medical schools in the country in response to the MEPI call for proposal. The MESAU consortium includes both a new school, Busitema University, and a private school, Kampala International University. In Ethiopia, the oldest school in the country, Addis Ababa University (AAU), invited medical schools to partner in their application for the MEPI award. AAU ultimately chose partners who expressed an interest in working together and had common challenges to address. In Nigeria, the University of Ibadan (a well-established medical school) formed the consortium with partner schools already working in the areas of HIV training and research. For these schools, the MEPI grant supplied the impetus to expand the scope of their collaboration to include medical education. In Zambia, the oldest and only public medical school in the country for decades, the University of Zambia (UNZA), was already providing faculty and administrative leadership for two new schools (one public and one private) prior to being awarded the MEPI grant. Shortly after receiving the grant, UNZA formed a consortium with these two new schools along with the one other medical school in country, leading to a national consortium. Table 1 provides more details on the history, size, and characteristics of each school in the four consortia.

### Establishing common goals

Each of the consortia leveraged MEPI funding to convene initial planning meetings of all medical school partners. The goals of each consortium that emerged were heavily influenced by national health priorities and by the three overarching goals of the MEPI grant, which include enhancing the capacity of medical schools to increase the quality and quantity of physicians, the retention of graduates in-country, and the research capacity of medical schools. Forming a unified voice to engage and influence government emerged as a priority goal for all schools. Given the scarcity of resources for training programmes, schools felt these partnerships would provide a vehicle to share infrastructure, faculty, and learning material efficiently and creatively. In Zambia, Uganda, and Ethiopia, the consortia have prioritized supporting new schools by creating a pipeline for faculty and sharing learning materials. All of the consortia have worked to improve the quality and consistency of education across the country by establishing national standards for training and creating opportunities to share effective curricula, best practices, and lessons learned.

#### Delivering high-quality education

The MEPI consortia are raising the bar for quality medical education by bringing schools together to establish national standards and share best practices. One such example is how the MESAU consortium brought their schools together to reach consensus on nine core competencies for undergraduate medical education in the country. These competencies are based specifically on their national health needs and form the foundation for the development of competency-based curricula for all training programmes in the country. Such efforts seek to ensure that all schools, regardless of age or resources, provide consistent and high-quality education.

The consortia have also provided an opportunity for schools across the country to examine, compare, evaluate, and, in some cases, disseminate education strategies. In Uganda, the consortium is capitalizing on the variability of community-based education (CBE) at the different schools. All schools use community rotations to nurture a sense of social accountability and improve graduate retention, yet they differ in their curriculum and duration. The consortium is conducting a multi-site analysis of community-based education programmes to determine which experiences achieve the desired outcomes. This evidence-based approach to reforming CBE will ensure that all schools in Uganda are able to provide a high-quality community experience for their students. Similarly, in Nigeria, the University of Jos, has implemented a new community-based experience, using district hospitals for clinical rotations. These rotations provide students with more hands-on opportunities to learn and practice clinical skills. Sharing the early success stories of this programme through the consortium has motivated other schools, and even the Ministry of Health, to incorporate this strategy of sending students out for rotations to offload crowded tertiary hospitals and improve the learning experience.

#### Sharing limited resources

The schools engaged in these consortia are redefining success by prioritizing national outcomes over school outcomes, triggering a paradigm shift in the country. Rather than focusing singularly on their institutional achievements, they are working towards national goals, and sharing and distributing in-country resources to collectively achieve a robust national health workforce. Where schools once competed for faculty and infrastructure, the consortia have found creative ways to pool and distribute resources efficiently. In Nigeria, for example, the University of Ibadan offers a unique course in reproductive health. Audiovisual training material was recorded and shared across the consortium in order to replicate this programme at other universities. Partner schools work in similar contexts so they were able to use the material with only minimal changes based on local cultural differences.

Historically, interactions and sharing of resources between public and private medical schools was limited, but the consortia have redefined these relationships. In Zambia, private schools have been invited to use the skills lab at UNZA and were also offered guidance and support in procuring their own skills lab equipment. Faculty exchanges, in response to countrywide shortages of basic science faculty, have connected public and private medical schools in Uganda and Zambia. These exchanges send faculty to teach courses, supervise students on clinical wards, and serve as external examiners. In Ethiopia, while the consortium consists of only four of the country’s twenty-six schools, it has spurred the formation of a national network of schools engaging in faculty exchanges across the country.

Research programmes in African medical schools have typically relied on northern partners for resources and expertise, but the consortia are providing new models of support. Each of the consortia are investing in ways to leverage and share the expertise of in-country research partners and improve efficiencies in research support systems. The MESAU consortium is developing an electronic IRB system that will ultimately enable schools to share the case load, making research support more efficient and increasing the research output at each institution. The partnership has also leveraged grant funding to provide a number of seed research grants to stimulate faculty and student-driven research across the country. Similarly, the consortia in Nigeria, Ethiopia, and Zambia are leveraging the experience of schools with strong research programmes to assist partner schools in setting up or strengthening IRBs and provide research training to faculty.

#### Supporting new schools

With limited in-country resources for training (such as faculty and clinical sites), new medical schools, particularly private schools, would previously have been seen as competition to older, more established schools. The consortia within the MEPI network demonstrate a shift in focus where schools are working together with the government to meet national health workforce needs. Each of the consortia is led by the oldest medical school in the country, which has embraced new medical schools (both public and private) as partners. Having trained physicians for decades, older schools have built strong relationships with their national governments and two schools even leveraged this relationship to advocate for the opening of new public medical schools. In Zambia, only one medical school had trained doctors for decades, until the leadership of the consortium convinced the government to open a new public medical school at Copperbelt University. In Uganda, government plans to fund a new medical school at Busitema University had stalled years before the consortium was established. The unified voice of the MESAU consortium convinced the government to open the new school. In forming these partnerships, the established schools provided the necessary assurance to the government that new schools would be supported and resourced to succeed.

Support for these new schools includes providing resources and helping to establish capacity in medical education. In Ethiopia, the two newer schools struggle with inadequate budgets, poor Internet connections, and limited access to learning materials for the growing student body. In response, AAU has secured eLearning resources for them, including eGranary, an Intranet-based repository that stores teaching materials and medical resources locally. AAU has also purchased electronic tablets loaded with medical textbooks for students across the consortium. The provision of these resources required some complex logistical manoeuvering as well as significant financial input from AAU. Similarly, the other three consortia are providing educational materials and eLearning resources to new schools in their consortium.

In all of the consortia, the older schools are providing a pipeline for faculty and leadership for these new institutions through postgraduate education. The Zambian consortium responded to a critical shortage of basic sciences faculty by creating Master’s-level programmes and training sponsored fellows from Lusaka Apex Medical University (a new private medical school) and Copperbelt University School of Medicine (a new public medical school). Sponsored graduates commit to return to their institutions as junior faculty. In Ethiopia, AAU is the primary training site in the country for all post-graduate programmes. With MEPI funding, AAU has expanded these graduate programmes, training specialists across the disciplines who will go on to be leaders and faculty at the dozen new schools that have opened in recent years. Moreover, AAU has developed a gender promotion programme specifically targeted at recruiting, retaining, and grooming females to become physician leaders. One of the graduates of this programme has already been appointed as Dean of the School of Medicine in a partner school. Contrary to the belief that older schools are less flexible and innovative, the consortia in MEPI are an example of how established schools are leveraging their experience and resources to creatively support new schools. If funding had gone directly to newer schools, such partnerships would have been unlikely and new schools would not have had this critical support.

### Implications for national health workforce planning

The consortia have strengthened the relationship between academia and policy-makers leading to more informed health workforce planning. The unified voice of medical schools in-country, anchored in the credibility of established institutions, has a strong influence on national health policy. Governments have leveraged this new platform to examine health workforce issues of national significance. For example, in Nigeria, the consortium is conducting a study with the Medical and Dental Council Ministry to examine current compulsory service policies. Their findings will ultimately advise the Council how to strengthen licensing requirements to improve the in-country health workforce geographical distribution. Similarly, the Ugandan consortium is conducting a government-commissioned study to examine student recruitment over the last 10 years. The findings will aid a reform of the medical school central admissions criteria. In Zambia, the consortium is working with the government to map out training programmes to avoid duplication and improve efficiencies. For example, the government and schools have agreed that diploma-level training in emergency medicine will only be provided by the private medical school Lusaka Apex and Master’s-level training in emergency medicine will be provided at the established medical school, UNZA. Historically, the heavy reliance on north-south partnerships has meant that medical school research was heavily influenced by external funders. With national consortia in place, medical schools in these four countries are now working with their governments to develop locally-relevant research agendas. The results of their research will in turn strengthen the health system through evidence-based policy changes.

### Challenges and critical success factors

The consortia all faced challenges in transforming the cultures within their institutions (to work with partner schools) and in executing collaborative programmes. Universities are competitive and often ranked against each other. It therefore took time to overcome the historical culture of individualism among the faculty and institutional leaders. Faculty and leaders within each school needed to be convinced that they should share resources to meet the needs of the national health workforce. In some cases, policies to support collaboration across institutions did not even exist. For example, in Ethiopia, AAU needed to develop new policies to facilitate the transfer of textbooks that were purchased for a partner university. Another challenge the consortia faced is the uneven pace of work at different schools. Progress on collaborative work was sometimes stymied by differences in institutional cultures, hierarchies, or approval processes.

Despite these barriers, schools have managed to work productively together even in the early years of their collaboration. The leaders attribute their success to both the MEPI grant and effective management of the partnership. The rigor and the focus of the grant brought partners together with a sense of purpose and common goals. MEPI’s thematic focus on health workforce issues of national relevance provided a platform to discuss common issues and the grant brought resources to develop solutions. The funding that MEPI provided supported interactions between schools such as site visits, meetings, and videoconferences, enabling the development of personal relationships. These interactions helped to overcome individualistic tendencies and encouraged work across institutions. Institutional relations were strengthened by engaging leadership at all levels. Senior leaders of the university, deans of medical schools, and department heads were all involved in decisions, discussions, and planning of consortia activities. This approach has enabled the spirit of collaboration to go beyond grant activities, permeating the culture across each institution.

### Sustainability

**Figure 1:**
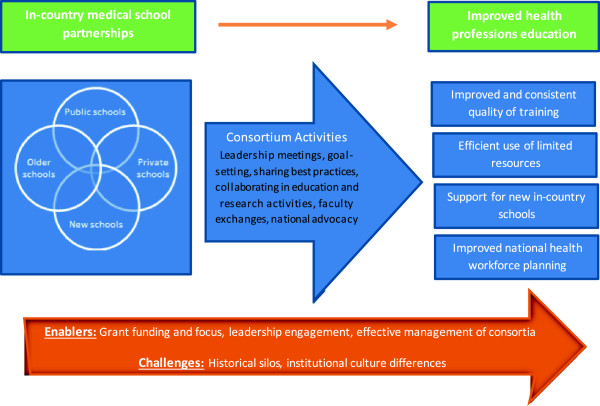
**Conceptual framework for in-country partnerships.** Model linking in-country partnerships with improved health professions education.

North-south partnerships need to balance the requirements of short-term grants that often support them with long-term goals for the country. In comparison, the south-south partnerships represented by these four consortia are working towards long-term health workforce goals and have already garnered local support for sustaining their efforts beyond the grant period. The leadership of each consortium has institutionalized the partnership by highlighting tangible benefits of collaboration and engaging senior leaders of the universities. Many of the activities prioritized in the first years reaped immediate results (such as sharing textbooks and faculty across schools). Such efforts have been so successful in demonstrating the value of working together that each of the consortia is expected to continue with local or other resources beyond the life of the MEPI grant. The MESAU consortium has already established a virtual presence with a web site, newsletter, and online communication platform which is expected to continue long past the MEPI grant period. In Nigeria, the consortium has already jointly applied for several other grants to strengthen and expand their work together. Similarly, the schools in Zambia and in Ethiopia have plans to continue working as a consortium to improve health workforce training.These early findings suggest that the model of south-south partnership may prove to overcome the challenges of north-south partnerships while still providing critical support to the growth and transformation of health professions’ education in Africa. Figure 1 is a conceptual framework derived from the findings of this study illustrating the link between these in-country partnerships and their influence on health professions’ education implementation and planning. As funders and stakeholders continue to invest in scaling-up the quality and quantity of health professionals in developing countries, this study highlights the value of investing in in-country partnerships. Factors that enable these partnerships to flourish should be noted and challenges should be mitigated in order to allow for strong, sustainable, mutually-beneficial partnerships.

### Limitations

Four of the authors (NS, MD, YM and DO) are closely involved in the implementation of the MEPI grant, participated as interviewees as well as in the development of the manuscript which may have led to more positive reporting and overstatement of results, than if there was an independent evaluation. Authors ZT, EM and HW also had multiple roles including interviewing, coding, analyzing and developing the manuscript which may also have led to researcher bias in the results of this study. Other limitations of this study include the small sample size as well as the fact that other stakeholders and participants were not included in this phase of the study.

## Conclusions

MEPI has clearly stimulated a paradigm shift in medical education in Africa by supporting south-south collaborations. Partnerships between medical schools, particularly public and private medical schools in-country, are innovative even by global standards. The medical schools in these consortia have been able to pool and mobilize resources creatively, standardize and generate locally-relevant curricula based on best-practices, and provide critical support to new schools. Unlike partnerships with northern schools, the consortia are driven by local needs and are more likely to be sustained beyond the grant period. These partnerships have given medical schools a stronger role in the health system to work with governments and influence health workforce planning. Future funding aimed at strengthening health professions education should prioritize such south-south partnerships to optimize outcomes from education investment.

## Authors’ information

ZM is an associate professor of medicine and of health policy at the George Washington University School of Medicine and Health Sciences, Washington DC, USA.

EKM is Director of the MEPI Coordinating Centre at the African Centre for Global Health and Social Transformation, Kampala, Uganda.

HW is a research associate for the MEPI Coordinating Centre in the Department of Health Policy at the George Washington University, Washington DC, USA.

MD is an associate professor of pediatric surgery and acting Vice President for the College of Health Sciences, Addis Ababa University, Addis Ababa, Ethiopia.

YM is Dean of the University of Zambia School of Medicine, Lusaka, Zambia.

DO is Former Dean, Faculty Basic Medical Science, College of Medicine, University of Ibadan, Ibadan, Nigeria.

NS is a professor of medicine and the Principal (Head) of Makerere University College of Health Sciences, Kampala, Uganda.

## Abbreviations

AAU: Addis Ababa University
CHEER: Collaboration for health equity in education and research
CBE: Community-based education
CONSAMS: Consortium of new southern African medical schools
IRB: Institutional review board
MEPI: Medical education partnership initiative
MEPIN: Medical education partnership initiative in Nigeria
MESAU: Medical education for equitable services to all Ugandans
PI: Principal investigator
UNZA: University of Zambia.
